# P25 Impact of infection specialist advice on the assessment and clinical outcomes of patients referred but not taken on to an outpatient parenteral antimicrobial therapy (OPAT) service

**DOI:** 10.1093/jacamr/dlaf230.032

**Published:** 2025-12-04

**Authors:** Mary Rimbi, Simon Pybus, Andrew Seaton

**Affiliations:** NHS Greater Glasgow and Clyde, Glasgow, UK; NHS Greater Glasgow and Clyde, Glasgow, UK; NHS Greater Glasgow and Clyde, Glasgow, UK

## Abstract

**Background:**

Whilst the impact of OPAT IV and complex-oral treatment episodes is well described, the wider impact of OPAT service assessment of patients referred to but not taken on has not been well described. This study explores antimicrobial stewardship (AMS) opportunities, clinical impact and patient outcomes in those referred to OPAT but not taken on in a large tertiary centre.

**Methods:**

All OPAT referrals that were screened but not taken on by the NHS Greater Glasgow and Clyde (NHSGGC) OPAT service over a 3 year period (2022 -2024) were reviewed. Those that were advised that specialist OPAT follow up was not required because an oral antibiotic option which did not require close monitoring were analysed for reason for referral, duration of antibiotic prescribed and patient outcome (readmission and mortality) at 30 days.

**Results:**

A total of 5024 patients were referred to OPAT during the study period and 1467 (29.2%) were not taken onto the service. 517 (35%) of these were given advice to switch to an oral antibiotic option that did not require specific OPAT monitoring. These included referrals for bone and joint, 238 (46%), complex intra-abdominal, 132 (25.5%), urinary tract, 27 (5.2%), *Staphylococcus aureus* bacteraemia or other blood stream infections, 21 (4%), respiratory 20 (3.9%), skin and soft tissue, 17 (3.3%), endovascular, 16 (3.1%) and endocarditis, 9 (1.7%) infections. ‘Oral recommended’ resulted in a total of 13 909 days of oral therapy at home over three years. Many of these patients were followed up separately in an infectious disease or other specialty clinic. Amongst the ‘oral recommended’ group, 30 day mortality was 3.9% (20/517) and 30 day re admission rate from date oral antibiotic advice was given was 17.2% (86/498). The majority of the readmissions were unplanned (86% or 74/86) and of these 43.2% (32/74) were related to the infection for which oral antibiotics were recommended.

**Conclusions:**

This data highlights the central role of AMS in OPAT gate keeping and the importance of measuring the unseen and wider healthcare service impact of OPAT. Assuming those referred had remained on IV therapy then the equivalent of 38 years of inpatient or OPAT IV days have been avoided. A small proportion of patients, 6.2% overall required readmission for treatment of the infection following switch to unsupervised oral therapy.

